# Mucosal-associated invariant T cells repress group 2 innate lymphoid cells in *Alternaria alternata*-induced model of allergic airway inflammation

**DOI:** 10.3389/fimmu.2022.1005226

**Published:** 2022-11-15

**Authors:** Yasuo Shimizu, Yukiko Horigane-Konakai, Yoshii Ishii, Chie Sugimoto, Hiroshi Wakao

**Affiliations:** ^1^ Department of Pulmonary Medicine and Clinical Immunology, Dokkyo Medical University, Mibu, Tochigi, Japan; ^2^ Respiratory Endoscopy Center, Dokkyo Medical University Hospital, Mibu, Tochigi, Japan; ^3^ Host Defense Division, Research Center for Advanced Medical Science, Dokkyo Medical University, Mibu, Tochigi, Japan

**Keywords:** mucosal-associated invariant T (MAIT) cells, group 2 innate lymphoid cells (ILC2s), allergic airway inflammation, Interferon γ (IFN-γ), mice rich in MAIT cells, *Alternaria alternata*, immunocompromised mice, MAIT1

## Abstract

Mucosal-associated invariant T (MAIT) cells, a blossoming member of the innate-like T cells, play a pivotal role in host defense through engaging the mucosal immunity. Although it has been suggested that MAIT cells are somehow implicated in the allergic airway inflammation mediated by group 2 innate lymphoid cells (ILC2s) such as asthma, the precise role(s) of MAIT cells in such inflammation has remained elusive. To explore the possible roles of MAIT cells in the inflammation, we examined whether MAIT cells suppressed the production of T helper (Th) 2 and inflammatory cytokines from ILC2s, and constrained the proliferation of ILC2s, both of which are prerequisite for airway inflammation. Given that laboratory mice are poor at MAIT cells, a novel mouse line rich in MAIT cells was used. We found that mice rich in MAIT cells showed alleviated airway inflammation as evidenced by reduced infiltration of the immune cells and hyperplasia in goblet cells in the lung concomitant with compromised production of Th2 and inflammatory cytokines, while wild type mice exhibited severe inflammation upon challenge with the fungal extracts. *In vitro* coculture experiments using purified ILC2s and MAIT cells unrevealed that cytokine-stimulated MAIT cells suppressed ILC2s to produce the cytokines as well as to proliferate most likely *via* production of IFN-γ. Furthermore, reconstitution of the allergic airway inflammation in the highly immunocompromised mice showed that ILC2-mediated inflammation was alleviated in mice that received MAIT cells along with ILC2s. We concluded that MAIT cells played a crucial role in suppressing the cytokine-producing capacity of ILC2s and ILC2 proliferation, that ultimately led to decrease in the allergic airway inflammation.

The results open up a novel therapeutic horizon in ILC2-mediated inflammatory diseases by modulating MAIT cell activity.

## Introduction

Allergic airway inflammation, such as asthma and allergic rhinitis, affects more than 300 million individuals and represents a worldwide health concern (1). The cause of morbidity has been attributed to a biased response towards a type 2 immune response that engages both innate and adaptive immune cells (2). T helper (Th) 2 cells in the adaptive branch and group 2 innate lymphoid cells (ILC2s) in the innate branch are essential for directing a type 2 immune response (3, 4). Due to the important roles attributed to ILC2s in host defense, inflammation, and tissue repair, many studies have shown that they are critical for inducing allergic airway inflammation (5). ILC2s are activated by inflammatory alarmins, such as interleukin (IL)-25, thymic stromal lymphopoietin, and IL-33. This activation, in turn, triggers the production of type 2 cytokines, such as IL-5, IL-9, and IL-13, concomitant with the expansion of ILC2s *per se*, which further aggravates the disease (6). IL-5 is required for the maturation, survival, and degranulation of eosinophils, while IL-9 promotes the proliferation of ILC2s and their homeostasis together with mast cell proliferation. IL-13 is responsible for the metaplasia or hyperplasia of goblet cells, mucus production, and the proliferation of bronchial smooth muscle cells in airway inflammation (7–9). In contrast, limited information is currently available on cells other than innate cells that counteract ILC2s *in vivo*. Since ILC2s have pivotal functions at the mucosal boundary, it is reasonable to assume that cells that are abundant at the mucosal site may influence the activity of ILC2s. Mucosal-associated invariant T (MAIT) cells belong to an emerging family of innate-like T cells, such as iNKT cells and γδT cells (10). Innate-like T cells bridge innate and adaptive immunity, and, thus, have been implicated in airway inflammation (11, 12). However, the role of MAIT cells in human airway inflammation remains unclear. Furthermore, regardless of the abundance of MAIT cells in humans, their rarity in laboratory mice has hindered the *in vivo* study of MAIT cells. Therefore, we generated a novel mouse line rich in MAIT cells to overcome this inherent issue. This mouse was generated with induced pluripotent cells (iPSCs) from murine MAIT cells, harboring rearranged *Trav1-Traj 33*, specific for MAIT cell T cell receptor (TCR) α in the allele, and designated as the Vα19 mouse (13). We herein report the impact of MAIT cells on ILC2-mediated allergic airway inflammation, and show that MAIT cells mitigated airway inflammation by repressing cytokine production by and the proliferation of ILC2s both *in vitro* and *in vivo*.

## Materials and methods

### Generation of Vα19 mice

Chimeric mice were generated by injecting MAIT-iPSC clone L7-1 into ICR 8 cell-embryo (14, 15). Resultant chimeric mice were crossed with C57BL/6NJcl females (CLEA Japan), and pups were screened for germline transmission of the rearranged locus for *Trav1-Traj33* using the sample from ear punching by PCR with the primer sets (Va19 F 5’-TCAACTGCACATACAGCACCTC-3’ and Ja33 R 5’-CATGCATTATTCAGCCAGTGCCTTCT-3’). Offspring of the germline-transmitted chimeric mice were crossed to obtain *Trav1-Traj33* homozygous male mice. The mice were then crossed with C57BL/6NJcl females (CLEA Japan). Resulting hemizygous mice harboring *Trav1-Traj33 *were designated Vα19 mice and used throughout the study.

### Mice and *Alternaria alternate* challenge

C57BL/6NJcl wild-type (WT) and NOD/Shi-scid, IL2Rγ KO (NOG) mice purchased from the breeder (CLEA Japan) and Vα19 mice were housed under the specific pathogen-free conditions at our animal facility. Female mice between 8-12 weeks old were used in experiments. All animal experiments were approved by the Internal Animal Ethics Committee at Dokkyo Medical University (permission number 1243). Mice were challenged with an *A. alternata* extract (ITEA Inc. Tokyo, 10 μg in 50 μl phosphate-buffered saline (PBS)) *via* intranasal instillation on days 0, 3, and 6 under anesthesia with isoflurane, while the control mice received intranasal instillation of PBS (16). Mice were euthanized by injecting the anesthesia (intraperitoneal injection of pentobarbital (100mg/kg body weight) at the indicated time points, and subjected to preparation of bronchoalveolar lavage fluid (BALF) and lung tissues.

### Cell preparation

To obtain BALF, the lungs were washed with 0.6 ml of PBS twice with a 18G plastic cannula-connected 1-ml syringe. The BALF was separated into liquid and cells by centrifugation at 400×g for 15 min at 4°C. The fluid portion was stored at -80°C for later cytokine quantification assays (see below). The cells were suspended in FACS buffer for cell count and for flow cytometric analysis. Lung immune cells were isolated from the tissues after BALF collection as described previously with some modifications (16). Briefly, the tissues were placed into a GentleMACS C-tube (Miltenyi Biotec) and cut into approximately 1-mm^3^ pieces. Three milliliters of tissue digestion solution (50μg/ml Liberase TM (Sigma-Aldrich), 10µg/ml DNase I (Sigma-Aldrich) in RPMI1640) was added per tissues from single animal and then incubated at 37°C for 45 min under gentle shaking. Suspensions were further homogenized using the GentleMACS dissociator (Miltenyi Biotec) with the program: m_lung_02_01 followed by passing through a 100µm-MACS Smart strainer. Cells were pelleted down and treated with 1× ammonium-potassium-chloride (ACK) lysing solution to remove the erythrocytes. After washing with Hanks’ Balanced Salt Solution (HBSS) supplemented with 2% FBS and 0.01% sodium bicarbonate, the cells were suspended with 30% Percoll in HBSS and centrifuged at 400×*g* for 30 min. Resulting cells were recovered from the bottom of the tube, washed with HBBS, and suspended in FACS buffer or 10% FBS-containing PRMI (R10).

Single cell suspension from the spleen was prepared by mashing the tissues through a 40-μm mesh cell strainer with a syringe plunger. Single cells were suspended in R10 supplemented with 10 mM HEPES pH 7.0, 0.1mM 2-mercaptoethanol, and 100 IU/ml of penicillin/100 μg/ml streptomycin (referred as cR10) and spun down at 400×*g* for 4 min. The cell pellet was suspended in sterile ice-cold MilliQ water for 15 sec to lyse erythrocytes and immediately neutralized with an equal volume of 2× PBS containing 4% FBS. After centrifugation, cells were resuspended in cR10.

Cell count was acquired on MACSQuant analyzer in FACS buffer containing 7-AAD.

### Histological analysis

Lungs were fixed with formaldehyde neutral buffer solution (10% (v/v)) for 48 h and then embedded in paraffin. Tissue samples were sliced at a thickness of 2 μm and stained with hematoxylin-eosin (HE) and Periodic Acid-Schiff (PAS). For immunofluorescence, 4 μm-thickness tissue sections were subjected to antigen retrieval with 0.05% Tris/EDTA buffer (10 mM Tris-HCl, pH9.0 and 1 mM EDTA) at 121°C for 20 min in an autoclave. Tissue sections were incubated in blocking buffer (20mM Tris-HCl, pH7.4 containing 1% BSA) at 4°C for overnight, followed by incubation with the anti-GATA-3 antibody (1:1000, Nichirei) to identify ILC2 and/or anti-CD3 (1:1000, Nichirei) to stain MAIT cells, respectively, at 4°C for overnight. After washing with tris-buffered saline (TBS) three times, the tissue samples were incubated with secondary antibodies; Alexa Fluor 488-conjugated goat anti-rabbit IgG (H+L) (Thermo Fischer) (1:1000, diluted in antibody diluent (Dako)) for CD3 or Alexa Fluor 568-conjugated goat anti-mouse IgG (H+L) (Thermo Fischer) (1:1000, diluted in antibody diluent) for GATA3 at 4°C for overnight. After washing with TBS three times, the samples were mounted with SlowFade Diamond Antifade Mountant with DAPI (Thermo Fischer).

All images such as HE, PAS and immunofluorescent staining were obtained by Mantra2 Quantitative pathology workstation (Akoya Biosciences). The quantification of PAS-positive areas within epithelial cells were performed with inForm (ver.2.6) in Mantra2. Adobe Photoshop (Adobe System) was used to assign the colors to three collected channels for immunofluorescence images (DAPI [blue or grey], Alexa Fluor 568 [magenta], and Alexa Fluor 488 [green]).

### Flow cytometric analysis

Up to 1×10^6^ cells were stained in 50 µl of FACS buffer with the relevant antibodies (1:100 dilution for each antibody) listed in **Table S1**. For ILC2 analysis, cell suspension was first stained with biotin labeled antibodies against the lineage markers, then stained with streptavidin-FITC and other antibodies. After washing with FACS buffer, cells were suspended in FACS buffer containing 7-AAD. Data were acquired with MACSQuant analyzer (Miltenyi Biotec) or Attune NxT Flow Cytometer (Thermo Fisher Scientific). Data was analyzed by FlowJo software (BD Biosciences).

### Preparation of ILC2s and MAIT cells

To isolate ILC2s from naïve mice, lung cell suspension was prepared from 20 female C57BL/6N mice as described above. Cell suspension was first stained with biotin labeled antibodies against the lineage markers, then stained with streptavidin-FITC followed by magnetic cell separation of FITC negative cells using anti-FITC microbeads and LS columns (Miltenyi Biotec). Resultant lineage marker negative cells were then stained with CD45-APC/Cy7, CD90.2-APC and ST2-PE, and subjected to cell sorting by BD FACS Aria II or BD FACS Jazz (BD Biosciences). CD45^+^lineage^-^CD90.2^+^ST2^+^ cells were sorted as ILC2s. Sorted ILC2s were further cultured in cR10 supplemented with 1×MEM non-essential amino acids, 1 mM sodium pyruvate, 50 μM 2-mercaptoethanol, 50 μg/mL gentamicin sulfate, and mouse IL-2 and IL-7 (10 ng/ml, respectively, R&D systems) for 14 days, and used in all the experiments unless otherwise indicated. For isolation of MAIT cells, lung and spleen cells were prepared from 10 Vα19 mice as described above and stained with APC-labeled 5-OP-RU loaded on mouse MR1 tetramer. MR1 tetramer positive cells were enriched with anti-APC microbeads followed by LS columns (Miltenyi Biotec). MAIT-enriched cells were further stained with TCRβ-APC/Cy7, B220-PE, F4/80-PE, and CD19-PE, and MAIT cells were sorted as B220^-^CD19^-^ F4/80^-^TCRβ^+^MR1 tetramer^+^ cells with BD FACS Jazz. Sorted MAIT cells were placed in cR10 containing IL-2 and IL-7 at 37°C 5% CO_2_ for overnight before all the experiments. Cytokine-activated MAIT (cyt-MAIT) were prepared by stimulating sorted MAIT cells with murine IL-12 (10 ng/ml, Fuji Film Wako), IL-15 (10 ng/ml, BioLegend) and IL-18 (10 ng/ml, R&D systems) for 18 h. Similarly, TCR-activated MAIT (5OR-MAIT) were prepared by stimulating sorted MAIT cells with 10 nM of 5-OP-RU for 18 h. 5-OP-RU was prepared from 5-A-RU (Toronto Research Chemicals) and methylglyoxal (Sigma-Aldrich) as previously described (14).

### Coculture experiments

Co-culture experiments consisted of two modes: direct, allowing direct cell-cell contact, and indirect, i.e., physically separating the two types of cells, but allowing the circulation of culture medium. In the former, ILC2s alone (5 × 10^3^ cells), naïve MAIT cells alone (5 × 10^3^ cells), ILC2s and naïve MAIT cells (ILC2+MAIT, 5 × 10^3^ cells for each), ILC2s and cyt-MAIT cells (ILC2+cyt-MAIT, 5 × 10^3^ cells for each), and ILC2s and 5OR-MAIT cells (ILC2+5OR-MAIT, 5 × 10^3^ cells for each) were cultured in the absence or presence of IL-33 (10 ng/ml, R&D systems) in the 96-well plate for 5 days. IL-33 was renewed on day 2 where appropriate. In the latter, the Transwell culture system (Corning, HTS transwell-96 permeable support 0.4 μm) was used, wherein ILC2s were spread at the bottom, while MAIT cells were placed in the upper chamber. The other conditions were same as the direct coculture.

The culture supernatant from day 2 and day 5 cocultures was subjected to multiplex cytokine assays (see below). The number of ILC2s and MAIT cells in the coculture was measured with MACSQuant flow cytometry (Miltenyi Biotec).

To track the cell division of ILC2s, ILC2s were labeled with 1 μM carboxyfluorescein diacetate succinimidyl ester (CFSE) in CFDA SE cell tracer kit (Invitrogen) according to the manufacturer’s instructions, then cocultured with MAIT cells. The number of cell divisions was estimated by measuring the fluorescence intensity of CFSE with MACSQuant flow cytometry.

The effects of IFN-γ and PD-L1 on the proliferation of ILC2s and MAIT cells were analyzed with anti-IFN-γ-neutralizing antibody (eBioscience) or anti-PD-L1-neutralizing antibody (BioXcell). Rat IgG1κ (10 μg/ml, eBioscience) or rat IgG2b isotype control (10 μg/ml, BioXcell) were used as isotype control.

### Measurement of cytokines

BALF or the supernatant from the *in vitro* coculture of cells was subjected to the quantification of cytokines with LEGENDplex mouse cytokine panel 2 (13 plex, GM-CSF, IFN-β, IL-1α, IL-1β, IL-3, IL-7, IL-11, IL-12p40, IL-12p70, IL-23, IL-27, IL-33, and TSLP) and the mouse Th cytokine panel (13 plex, IL-5, IL-13, IL-2, IL-6, IL-9, IL-10, IFN-γ, TNF-α, IL-17A, IL-17F, IL-4, IL-21, and IL-22) (BioLegend) according to the manufacturer’s instructions. The analysis was performed with MACSQuant flow cytometer.

### RNA-sequencing analysis

MAIT cells (CD19^-^TCRβ^+^CD44^+^mMR1-tet^+^) were sort-purified from the lungs of PBS-treated and *A. alternata*-challenged WT mice (WT day 0 and WT day 8, respectively) and of similarly treated Vα19 mice (Vα19 day 0 and Vα19 day 8, respectively) and subjected to a RNA-sequencing analysis as follows. RNA was prepared with TRIZOL Reagent (Thermo Fisher Scientific) followed by the RNA clean & concentrator column with DNaseI (Zymo Research). cDNA libraries were generated using the SMART-Seq v4 ultraLow Input RNA kit for sequencing (Takara Bio). The quality and quantity of RNA were assessed using the RNA 6000 pico kit (Agilent Technologies) and Bioanalyzer 2100 (Agilent Technologies). RNA sequencing was performed by Hiseq/NovaSeq (Illumina). Sequence reads were aligned to the reference genome sequence using HISAT2 (v2.0.1), the gene expression analysis was performed with HTSEQ (v0.6.1), and differentially expressed genes (DEG) were analyzed with Deseq2 (v1.6.3) (GENEWIZ, Azenta Life Sciences). Transcripts showing >2 or <0.5 fold changes with FDR (adjusted P value) <0.05 were considered to be DEG. Antibodies and reagents to detect MAIT cells were anti-CD19 (BioLegend), anti-TCRβ (BioLegend), anti-CD44 (BioLegend), and 5-OP-RU-loaded mouse MR1-Tetramer (mMR1-tet, NIH Tetramer Facility).

### Adoptive transfer experiments

Sort-purified MAIT cells (1.0 × 10^6^ cells each per mouse) and/or sort-purified and ILC2s precultured for 14 days (1.0 × 10^6^ cells per mouse) as described above were adoptively transferred into NOG mice *via* an intravenous injection (i.v.). IL-33 (0.5μg in 20 μl PBS per mouse) was intranasally administrated 3 h after the adoptive transfer followed by boosting on day 3 with the same dose. Mice were euthanized on day 6 and subjected to the analysis of BALF and lungs as described above.

### Statistical analysis

Statistical analyses between two groups were performed by an unpaired *t*-test with Welch’s correction. Two-way repeated measures ANOVA was used to analyze serial changes. One-way ANOVA followed by the Bonferroni multiple comparison test or Dunnett’s multiple comparison test was conducted for three or more groups. All statistical analyses were performed with GraphPad Prism Software version 9. *p<0.05 was considered to be significant.

## Results

### Vα19 mice showed mitigated allergic airway inflammation

**Figure 1:**
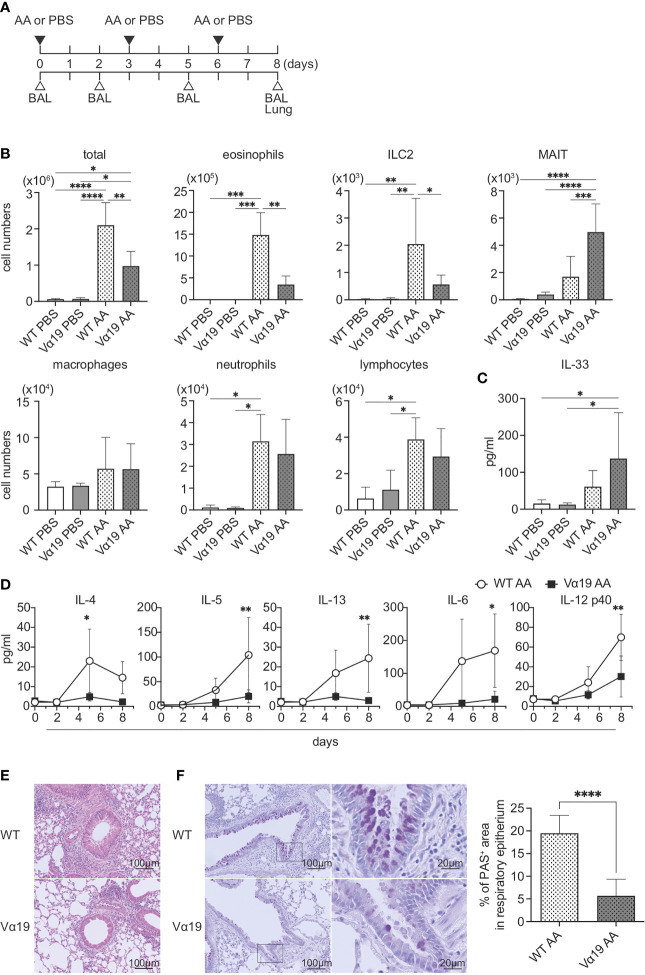
Mitigated lung inflammation in Vα19 mice upon the *A alternata* challenge **(A)** Schematic representation of the experiment. The challenge as well as the sampling schedule are indicated (AA: *A alternata* challenge, PBS: phosphate-buffered saline challenge) (upper panel), BAL: bronchoalveolar lavage, and the lungs (lower panel). *A alternata* extract (10 μg in 50 μl PBS) or PBS (50 μl) was intranasally challenged on day 0, 3 and 6. **(B)** Quantification of cell subsets in BAL fluid. The number of the indicated cell subsets in BAL fluid (BALF) sampled on day 8 are shown. WT PBS: wild-type mice challenged with PBS, Vα19 PBS: Vα19 mice challenged with PBS, WT AA: wild-type mice challenged with *A alternata*, Vα19 AA: Vα19 mice challenged with *A alternata.* Data are shown as mean ± SD (n=4 per group), *P<0.05, **P<0.01, ***P<0.001, ****P<0.0001. Data from two independent experiments are shown. **(C)** IL-33 production upon the *A alternata* challenge. IL-33 concentrations in BALF from the indicated mouse groups 1 h after the challenge are shown. Data are shown as mean ± SD (n=4 per group). The mouse grouping is the same as that in **(B)** Data from two independent experiments are shown. **(D)** Time course of cytokine production. Cytokine concentrations in BALF at the indicated day after the challenge in *A alternata*-challenged wild-type (WT AA) and Vα19 (Vα19 AA) mice. Data are shown as mean ± SD (n= 4 per group), *P<0.05, **P<0.01. Data from at least two independent experiments are shown. **(E, F)**. Histological analyses of WT and Vα19 mouse lungs. **(E)** Lung tissue sections stained with hematoxylin and eosin on day 8 after the *A alternata.* challenge. WT: wild-type mice, Vα19: Vα19 mice (left panels). The scale bar indicates 100 μm. **(F)** Same as described in E, except that the tissue sections were stained with PAS. Scale bars indicate 100 μm and 20 μm, respectively (left panel). Imaging of PAS stain was obtained with analysis software (inform, ver.2.6) equipped in Mantra2. The percentage of the PAS-positive area relative to the total surface of epithelial cells is shown as mean ± SD (n=4 per group) (right panel), ****P<0.0001. Data from at least five different views were used for measurement.

To examine the function of MAIT cells in allergic airway inflammation in relation to ILC2s *in vivo*, we assessed inflammation induced by *A. alternata* in wild-type (WT, C57BL/6) and Vα19 mice, as shown in **Figure 1A**. In the first step, BALF was analyzed (**Figure S1**). While the *A. alternata* challenge increased the total number of mononuclear cells in BALF in WT mice, that in Vα19 mice exhibited a smaller increase. Similar results were obtained for eosinophils and ILC2s. In contrast, the number of MAIT cells increased upon the *A. alternata* challenge in Vα19 mice, while a slightly smaller increase was noted in WT mice. It is noteworthy that while the frequency of MAIT cells in WT mouse lung rarely exceeded 3% of the total T cells, that in Vα19 mouse was superior to 35% (**Figure S2**) (13). In contrast, macrophage, neutrophil, and lymphocyte numbers did not significantly differ between WT and Vα19 mice (**Figure 1B**). This increase was specific to *A. alternata* because the challenge with PBS resulted in the negligible accumulation of all cell types examined, except for macrophages and lymphocytes, irrespective of the mouse strain (**Figure 1B**). Since the *A. alternata* challenge induces alarmin and triggers type 2 immunity, the production of IL-33 and type 2 cytokines was analyzed in BALF. IL-33 was detected as early as 1 h after the challenge in both mice, but little statistical difference was seen between Vα19 and WT mice (**Figure 1C**). The analysis of BALF further revealed that IL-4 levels peaked on day 5, followed by a decrease, whereas those of IL-5 and IL-13 continued to increase thereafter. Furthermore, inflammatory cytokines, such as IL-6 and IL-12p40, showed similar changes in both mice, but at markedly lower levels in Vα19 mice (**Figure 1D**). Moreover, the amount of IL-12p70 and IL-23 in BALF was below the detection limit (**Figure S3**). We also measured the other cytokines relevant to MAIT cell function such as IL-17A, IL-17F, IFN-γ, TNF-α, and GM-CSF. However, only low amount of GM-CSF could be detected on day 5 (data not shown). We then investigated whether these differences had an impact on lung tissue inflammation. HE staining revealed that the infiltration of inflammatory cells into peribronchiolar and perivascular connective tissues was significantly less in Vα19 mice than in WT mice. PAS staining showed that the metaplasia or hyperplasia of goblet cells was strongly mitigated in Vα19 mice (**Figures 1E, F**). The compromised production of type 2 and inflammatory cytokines reflected less severe airway inflammation in Vα19 mice (**Figures 1D–F**). Collectively, these results implied that the increase in MAIT cells alleviated allergic airway inflammation by suppressing the production of type 2 and inflammatory cytokines.

### Transcriptomes in MAIT cells

**Figure 2:**
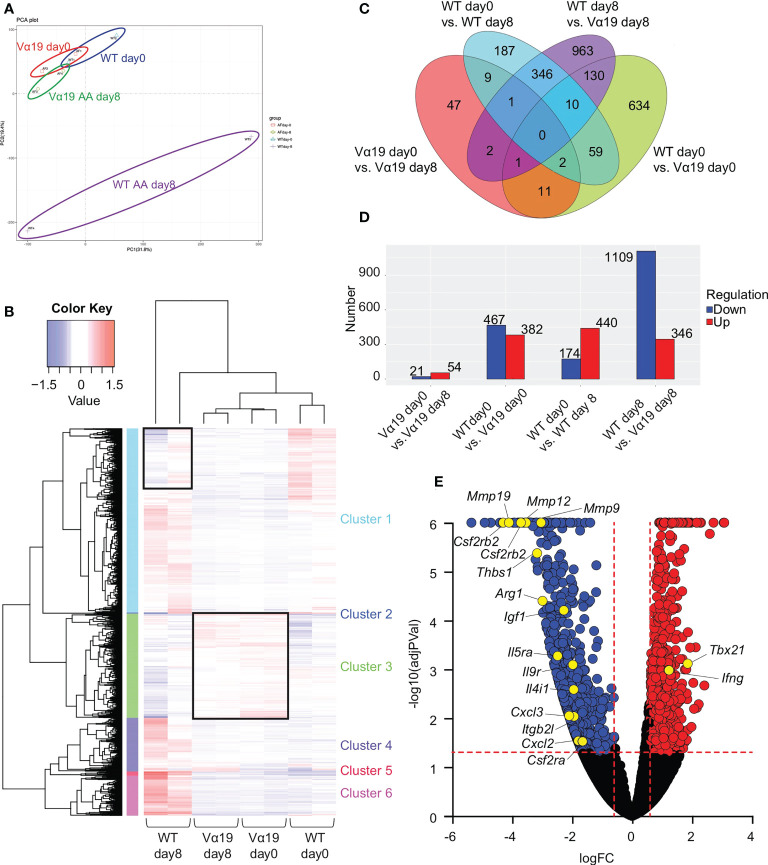
Transcriptome analysis of MAIT cells **(A)** Principal component analysis (PCA) Transcriptional data on MAIT cells from naïve wild-type mouse and Vα19 mouse lungs before the challenge (WT day 0 and Vα19 day 0), and those after the *A alternata.* challenge for 8 days (WT AA day 8 and Vα19 AA day 8) are plotted (n=2 per group). **(B)** Heatmap analysis Heatmap analyses of transcripts from *A alternata-*challenged wild-type and Vα19 mouse lung-derived MAIT cells (WT day 8 and Vα19 day 8, respectively), and naïve Vα19 and wild-type mouse lung-derived MAIT cells (Vα19 day 0 and WT day 0, respectively) are shown (n=2 per group). Relative expression levels are depicted in different colors. **(C)** Venn’s diagrams showing differentially expressed genes (DEG) common to the indicated groups. The number indicates the number of genes categorized in the indicated zone. **(D)** The number of DEG. The numbers of up- (red) and down-regulated genes (blue) between the designated populations are shown. **(E)** Volcano plot Volcano plot depicting DEG between MAIT cells in WT day 8 and those in Vα19 day 8. Genes that increased in the latter are indicated in red, while those that decreased are in blue. The genes potentially related to the inflammation suppressive functions and those relevant to MAIT1 gene signature are shown with yellow circles. X-axis: Log2 [fold change (FC)], Y-axis: -Log10(adjusted P value).

To elucidate the molecular pathways underlying the protective roles of MAIT cells in airway inflammation, a transcriptome analysis was performed on MAIT cells. The results of a principal component analysis (PCA) and heatmap analysis implied that MAIT cells from WT mice after the *A. alternata* challenge harbored a more distinct profile than those from other sources (**Figures 2A, B**). The *A. alternata* challenge induced a number of DEG, some of which were common between the indicated groups (**Figures 2C, D**). Since Vα19 mice showed less severe airway inflammation than WT mice, we focused on genes relevant to this difference. A volcano plot indicated that the expression of *Tbx21* and *Ifng* was higher in Vα19 mice than in WT mice upon the *A. alternata* challenge (**Figures 2E** and **Table S2**). In contrast, transcripts relevant to tissue remodeling, such as *Mmp9*, *Mmp12*, and *Mmp19*, were lower (17–19). Similarly, the suppression of transcripts related to inflammation, such as *Ccl3*, *Arg1*, *Il9r*, *Csf2ra*, *Csf2rb*, and *Csf2rb2*, concomitant with those important for tissue repair, including *Cxcl2*, *Thbs1*, *Igf1*, and *Itgb2l*, was observed (**Figures 2E** and **Table S2**) (20–24). These results indicated that MAIT cells in Vα19 mice highly expressed the transcripts for the Th1 response and weakly expressed genes relevant to inflammation, tissue remodeling and repair.

### MAIT cells suppressed the proliferation of and cytokine production by ILC2s

**Figure 3:**
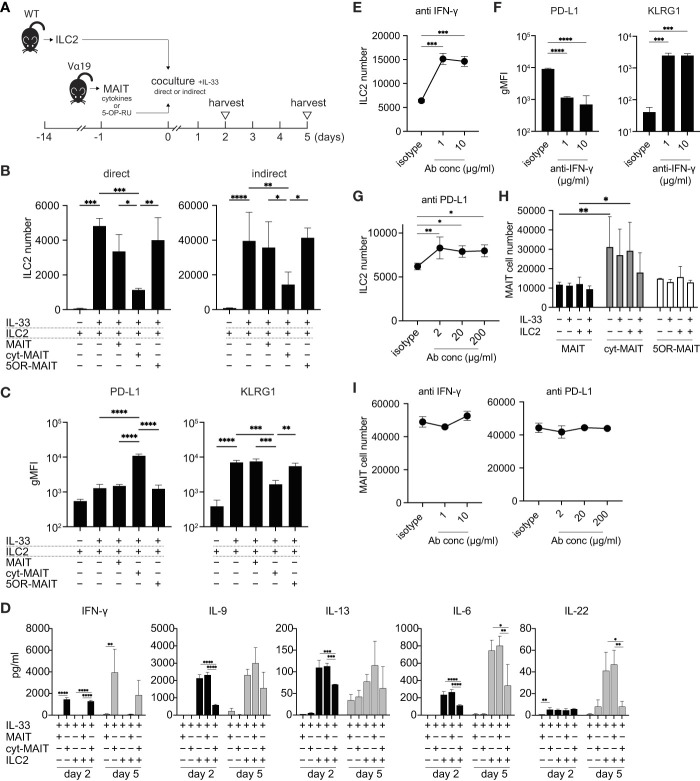
Compromised cytokine production by and proliferation of ILC2s by MAIT cells **(A)** Schematic representation of the experiment. ILC2s prepared from wild-type mice (5 × 10^3^ cells) and MAIT cells (5 × 10^3^ cells) from Vα19 mice that were sham-treated, treated with IL-12 (10 ng/ml), IL-15 (10 ng/ml), and IL-18 (10 ng/ml) (cytokines), or with 5-OP-RU (10 nM) for 18 h were subjected to cocultures in the 96 well Transwell culture system. The purity of isolated ILC2s and MAIT cells was more than 89% and 79%, respectively as judged by flowcytometry. On day 2, the culture supernatant was subjected to a cytokine assay, while other assays including the cytokine quantification were performed on day 5. **(B)** Suppression of ILC2 proliferation by cyt-MAIT cells. ILC2 cell numbers upon the coculture with the designated MAIT cells are indicated. MAIT: naïve MAIT cells, cyt-MAIT: IL-12, IL-15, and IL-18-stimulated MAIT cells, 5OR-MAIT: 5-OP-RU-stimulated MAIT cells. The direct culture allowed the contact between ILC2s and MAIT cells, while the indirect culture physically separated these cells in a transwell. Data are shown as mean ± SD (n=3), *P<0.05, **P<0.01, ***P<0.001, ****P<0.0001. Data from at least two independent experiments are shown. **(C)** Regulation of PD-L1 and KLRG1 in ILC2s. PD-L1 and KLRG1 expression in ILC2s after the indirect coculture described in **(B)** Data are shown as mean ± SD (n=3), **P<0.01, ***P<0.001, ****P<0.0001. Data from at least two independent experiments are shown. **(D)** Cytokine production by ILC2s and/or MAIT cells. Concentrations of the indicated cytokines from ILC2s, naïve MAIT cells (MAIT), cyt-MAIT cells (cyt-MAIT), and the coculture of ILC2s with naive MAIT cells or with cyt-MAIT cells for 2 days (bars filled with black) and 5 days (bars filled with grey) are shown. Data are mean ± SD (n=3). Data from two independent experiments are shown. **(E)** Release of the inhibition of ILC2 proliferation. ILC2 numbers in the indirect coculture with cyt-MAIT cells for 5 days in the presence of isotype control IgG (isotype) or the indicated amounts of IFN-γ-neutralizing antibody are shown. Data are mean ± SD (n=3), ***P<0.001. Culture conditions are the same as described in **(B)** Data from at least two independent experiments are shown. **(F)** Expression of PD-L1 and KLRG1 upon the neutralization of IFN-γ. This is the same as that described in E, except that the expression levels of PD-L1 and KLRG1 in ILC2s are depicted. Data are mean ± SD (n=3), ***P<0.001, ****P<0.0001. Data from two independent experiments are shown. **(G)** Release of PD-L1-mediated inhibition of ILC2 proliferation. This was the same as that described in E, except that the PD-L1-neutralizing antibody was used at the indicated concentrations. Data are mean ± SD (n=3), *P<0.05, **P<0.01. Data from at least two independent experiments are shown. **(H)** MAIT cell proliferation. Sort-purified MAIT cells (5 × 10^3^ cells) left untreated (naive MAIT), treated with IL-12/IL-15/IL-18 (cyt-MAIT), or 5-OP-RU (5OR-MAIT) were cocultured with or without ILC2s (5 × 10^3^ cell) in Transwell as described in **(A)** Resulting MAIT cell numbers in the absence or presence of IL-33 (10 ng/ml) for 5 days are shown. Data are mean ± SD (n=3), *P<0.05, **P<0.01. Data from at least two independent experiments are shown. **(I)** IFN-γ and PD-L1-independent proliferation of MAIT cells. The experiments are the same as E and G, except that cyt-MAIT cell numbers are measured. Data show the number of the cells after 5 days culture and are mean ± SD (n=3). Data from two independent experiments are shown.

Since the number of ILC2s was lower in Vα19 mice than in WT mice upon the *A. alternata* challenge and the number of MAIT cells inversely correlated with that of ILC2s in BALF accompanied by an elevated IL-33 level, we investigated whether MAIT cells interfered with the proliferation of ILC2s. To address the issue, ILC2s and MAIT cells were cocultured *in vitro* as shown (**Figures 3A** and **S4**). Two different culture modes were used to distinguish whether this interference was dependent on cell-cell contact (direct) or mediated by a soluble factor(s)(indirect). ILC2 proliferated upon the IL-33 stimulation (**Figure 3B**), which is consistent with previous findings (25). While the coculture with naïve MAIT cells purified from Vα19 mice resulted in few changes, IL-12-, IL-15-, and IL-18-stimulated MAIT cells (hereafter referred to as cyt-MAIT cells) that represent a TCR-independent activation suppressed the expansion of ILC2s irrespective of the culture mode. In contrast, 5-OP-RU (an agonist for MAIT cells)-stimulated MAIT cells (hereafter referred to as 5OR-MAIT cells) that represent a TCR-dependent activation did not (**Figure 3B**). Accordingly, the IL-33-dependent cell division of ILC2s was completely inhibited by cyt-MAIT cells (**Figure S5**). We then investigated whether cyt-MAIT cells affected the expression of the molecules pertinent to ILC2 proliferation and found the up-regulated expression of PD-L1 concomitant with the down-regulation of KLRG1 in the indirect coculture, whereas the IL-33-dependent up-regulation of PD-1, CD25, and Sca1 was not affected. Neither naïve MAIT cells nor 5OR-MAIT cells interfered with the expression of PD-L1 or KLRG1 (**Figures 3C**, **S6**) (26–28). In contrast, 5OR-MAIT cells appeared to repress the expression of ICOS and ICOS-L more in ILC2s than in cyt-MAIT cells (**Figure S6**). These results indicated that soluble factor(s) from cyt-MAIT cells were responsible for the repression of ILC2 expansion and the expression of these molecules. To obtain more detailed insights, the supernatants from different combinations of culture conditions were subjected to a multiplex cytokine analysis. IFN-γ was produced by cyt-MAIT cells, but not by naïve MAIT cells or ILC2s (**Figure 3D**). While the production of IFN-γ was not inhibited by ILC2s, IL-22 from ILC2s was compromised by cyt-MAIT cells on day 5 (**Figure 3D**). To establish whether IFN-γ was responsible for repressing the expansion of ILC2s and controlling the expression of PD-L1 and KLRG1, an IFN-γ-neutralizing antibody was added to the indirect coculture. A concentration as low as 1 μg/ml of the antibody derepressed the proliferative inhibition imposed by cyt-MAIT cells as well as the up- and down-regulation of PD-L1 and KLRG, respectively, in ILC2s (**Figures 3E, F**). Since the expression of PD-L1 was up-regulated in cyt-MAIT cells and signaling through PD-1-PD-L1 negatively regulated the proliferation of ILC2s, we investigated whether the blockade of PD-1 also alleviated the proliferative inhibition by cyt-MAIT cells (27). The addition of the PD-L1-neutralizing antibody, but not control IgG resulted in the resumption of ILC2 expansion (**Figure 3G**). To clarify the effects of ILC2s on MAIT cell proliferation, the expansion of naïve MAIT cells, cyt-MAIT cells, and 5OR-MAIT cells was examined. The results obtained showed that cyt-MAIT cells expanded autonomously, while other MAIT cells did not, irrespective of ILC2s and/or IL-33 (**Figure 3H**). Moreover, neither IFN-γ- nor PD-L1-neutralizing antibodies interfered with proliferation (**Figure 3I**). These results showed that the stimulation with IL-12, IL-15, and IL-18 promoted MAIT cell proliferation concomitant with IFN-γ production, resulting in the repression of cytokine production by ILC2s as well as their proliferation.

### Reconstitution of the repressive activity of MAIT cells over ILC2s in immuno-compromised mice

**Figure 4:**
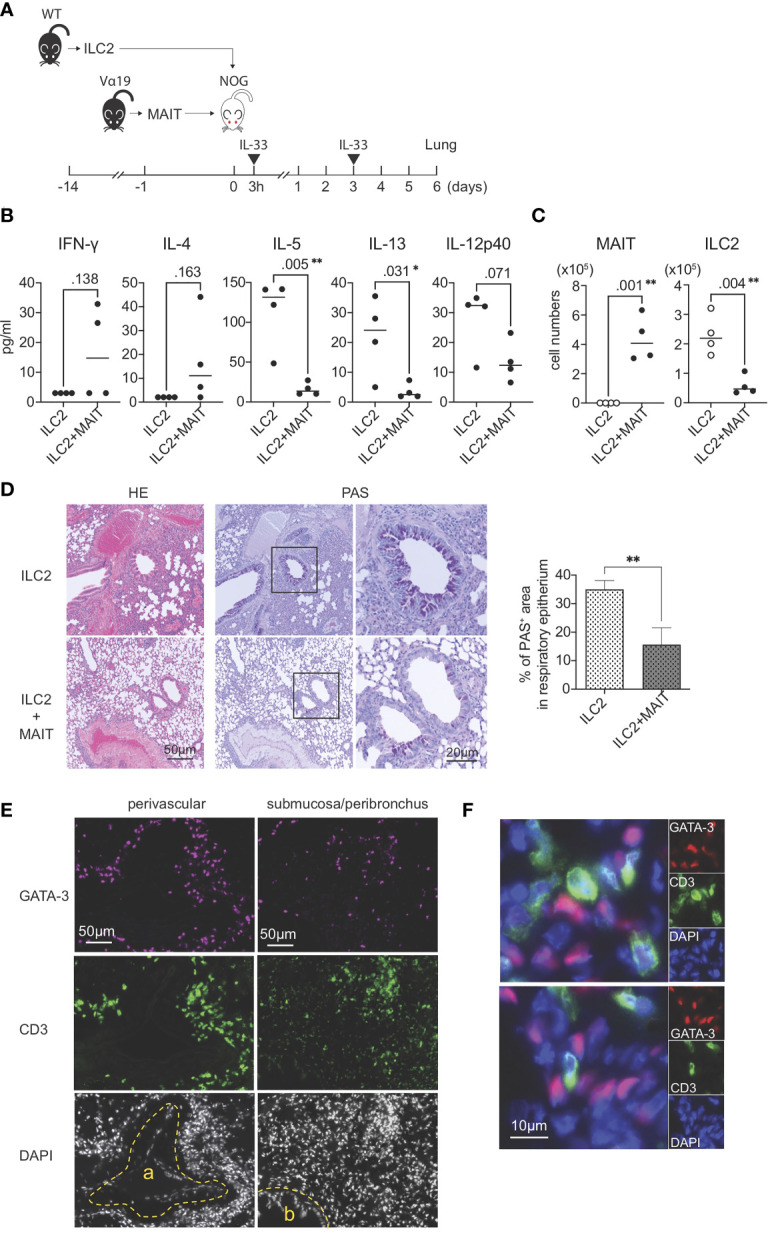
Reconstituted suppression of ILC2-mediated allergic airway inflammation by MAIT cells. **(A)** Schematic representation of the experiment. ILC2s (1.0 x 10^6^ cells/mouse, the purity >89% defined as CD90.2^+^ST2^+^ cells among CD45^+^lineage^-^ cells) alone from wild-type (WT) mice or in combination with sort-purified MAIT cells (1.0 x 10^6^ cells/mouse, the purity >79% defined as B220^-^CD19^-^F4/80^-^TCRβ^+^MR1 tetramer^+^ cells among lymphocytes) from Vα19 mice (Vα19) were adoptively transferred into NOG mice followed by an intranasal challenge with IL-33 (0.5μg in 20 μl PBS per mouse) as indicated. Sampling was performed on day 6. **(B)** Cytokines in BALF. The concentrations of the indicated cytokines in BALF from NOG mice received only ILC2s (ILC2) or both ILC2s and MAIT cells (ILC2+MAIT) are shown. Data are shown as mean ± SD (n=4), *P<0.05, **P<0.01. **(C)** Suppression of ILC2 accumulation in the lungs. The numbers of MAIT cells and ILC2s in the lungs of NOG mice received ILC2s only, and those received both ILC2s and MAIT cells are shown. MAIT (MAIT cells), ILC2 (ILC2s) Data show mean ± SD (n=4), **P<0.01. **(D)** MAIT cell-mediated mitigation of lung inflammation. HE and PAS staining of lung tissue sections from NOG mice received only ILC2s (ILC2, upper panels) and those received both ILC2s and MAIT cells (ILC2+MAIT, lower panels) are shown. Bars indicate 50 μm and 20 μm, respectively. In PAS staining, the rectangles indicate the enlarged regions shown on the right. Right panel shows the percentage of PAS^+^ area within the epithelial cells. **P<0.01. Representative data from at least 5 different areas are shown (n=3). **(E)** Localization of ILC2s and MAIT cells in the lungs. ILC2s and MAIT cells in the NOG mouse lungs upon adoptive transfer are identified with surrogate markers. ILC2s are shown as GATA3^+^ cells (red or magenta) and MAIT cells as CD3^+^ cells (green) within tissue sections representing the perivascular, submucosal, and peribronchial regions. a: artery, b: bronchus. Nuclei are stained with DAPI. Bars indicate 50 μm. Representative data from 4 mice are shown. **(F)** Juxtaposition of ILC2s and MAIT cells in the lungs. Representative superimposed images of GATA3-expressing cells (magenta) and those expressing CD3 (green), representing ILC2s and MAIT cells, respectively, in the NOG mouse lungs. Nuclei are stained with DAPI (blue). Bars indicate 10 μm. Representative data from 4 mice are shown.

The present results strongly suggested that MAIT cells *per se* suppressed the function and proliferation of ILC2s. To confirm this hypothesis, allergic airway inflammation was reconstituted with exogenous ILC2s alone or ILC2s together with MAIT cells in NOG mice devoid of immune cells in nature (**Figure 4A**). A multiplex cytokine analysis of BALF revealed that MAIT cells inhibited the production of IL-5, IL-13, and IL-12 p40 from ILC2s, but slightly increased that of IFN-γ and IL-4 (**Figure 4B**). Furthermore, the co-transfer of MAIT cells decreased the number of ILC2s in the lungs more than ILC2s alone and resulted in MAIT cell migration into the lungs (**Figure 4C**). The adoptive transfer of ILC2s alone followed by the IL-33 challenge resulted in typical allergic airway inflammation, as evidenced by the massive infiltration of immune cells, the hyperplasia or metaplasia of goblet cells, and the increased deposition of mucin concomitant with thickened smooth muscle cells in the airways. Immunohistochemical analysis uncovered that ILC2s tended to converge around the peribroncus, corroborating the results of HE staining (**Figures 4D** ILC2 and **Figure S7**). In marked contrast, the co-transfer of MAIT cells alleviated inflammation (**Figure 4D**, ILC2+MAIT). We then examined the localization of ILC2s and MAIT cells in the lungs. The results obtained showed that ILC2s and MAIT cells were juxtaposed in the submucosal compartment and around the artery (**Figures 4E–F**). The data demonstrated that ILC2s and MAIT cells colocalized in the lungs, thereby allowing MAIT cells to inhibit the functions of ILC2s.

## Discussion

The present results showed that MAIT cells, an emerging member of innate-like T cells, constrained both the function and proliferation of ILC2s *via* IFN-γ, which ultimately led to the suppression of eosinophilic airway inflammation. Innate-like T cells bridge innate and adaptive immunity, and, thus, play a pivotal role in inflammatory diseases, including asthma. However, the role of MAIT cells in asthma in humans remains unclear. Although increases in IL-17-producing MAIT cells in BALF from asthmatic patients have been correlated with disease exacerbation, MAIT cell numbers have been shown to decrease in the blood, sputum, and lung biopsies of patients (29, 30). Nevertheless, given their abundance in humans and importance as innate-like T cells, further studies are warranted to elucidate the role of MAIT cells in asthma. In this respect, murine model(s) will provide mechanistic insights into the possible role(s) of MAIT cells. However, MAIT cells are rare in the laboratory mouse strains currently available. The frequency of MAIT cells in mice relative to that in humans is less than 1/10~1/100, which has limited the elucidation of their function. To overcome this issue, we generated a novel mouse strain Vα19 mouse *via* iPSCs (13). Given that the frequency of MAIT cells in Vα19 mouse is quasi-equivalent or superior to that in humans, it allowed us to elucidate the roles of MAIT cells in an asthma model in relation to ILC2s.

While MAIT cells have been shown to suppress Th2 cytokine production by ILC2s, but not their proliferation, the present results demonstrated that MAIT cells suppressed both. The inability of MAIT cells prepared by Ye and colleagues to repress the proliferation of ILC2s may be due a qualitative difference in the MAIT cells used. Ye and colleagues used MAIT cells expanded with exogenous IL-7; however, the precise nature of the cells remains uncharacterized (31). In contrast, the present results indicated that MAIT cells stimulated with IL-12, IL-15, and IL-18, but not naïve MAIT cells from Vα19 mice, inhibited both cytokine production by and the proliferation of ILC2s *in vitro*. Moreover, 5-OP-RU-stimulated MAIT cells did not exert these effects, which implied that TCR-elicited signals are not essential for the inhibitory effects of these cells on ILC2s. Since MAIT cells in Vα19 mice were intact in terms of the ability to be activated and produce a plethora of cytokines (13), these results indicate that TCR-independent signal(s) are responsible for exerting the inhibitory activity of MAIT cells, which ultimately leads to the production of IFN-γ. Since IL-12 and IL-18 induce IFN-γ *via* Jak2/Tyk2-Stat4 and MyD88-IRAK-TRAF-6-NF-κB, respectively, further studies are warranted to investigate whether these signaling pathways are also responsible for IFN-γ production in cytokine-activated MAIT cells. Although the IL-4-induced 1 gene in MAIT cells is considered to represses the production of Th2 cytokines, such as IL-5 and IL-13, from ILC2s, the present results suggest that IFN-γ from cyt-MAIT cells compromise the production of a wider array of cytokines not confined to Th2 cytokines (31–33) (**Figure 3D**).

Our transcriptome data indicated that MAIT cells were biased to MAIT1 cells in Vα19 mice, which is supported by the intrinsically higher expression levels of *Tbx21* and *Ifng* in these mice than in WT mice irrespective of the *A. alternata* challenge (**Figure 2E** and **Table S2**, WT day 0 vs. Vα19 day 0). It is tempting to postulate that the niche required for MAIT cell maturation was saturated due to too many immature MAIT cells in Vα19 mouse, which in part reflected the difference in gene expression relative to that in C57BL/6 mouse (**Figure 2E**). While MAIT cells in C57BL/6 mouse lungs mostly comprise MAIT17 cells, those in Vα19 mouse are MAIT1-dominant, comprising immature MAIT and MAIT1 cells. Such a phenotype ultimately could lead to suppression of the airway inflammation concomitant with decline of the Th2-cytokines (**Figure 1**) (34). However, the rational for such MAIT1 bias has yet to be determined.

Regarding inflammation, MAIT cells *per se* may have alleviated airway inflammation by suppressing transcripts relevant to inflammation as well as tissue remodeling and repair (**Figure 2E** and **Table S2**, WT day 8 vs. Vα19 day 8) (17–24). These features, in turn, may have resulted in the repression of ILC2 differentiation and proliferation most likely *via* IFN-γ *in vivo*.

Although the present results showed that IFN-γ from MAIT cells was responsible for constraining the functions of ILC2s, molecules engaging in cell-cell contact may also contribute to the inhibitory activity of these cells on ILC2s. Accordingly, the compromised expansion of ILC2s correlated with the up-regulation of PD-L1 concomitant with the down-regulation of KLRG1 in ILC2s (**Figures 3C**). PD-L1 is induced in ILC2s upon an IL-33 challenge, and is a prerequisite for mounting an efficient Th2 immune response, such as worm expulsion, but is dispensable for Th2 cytokine production by ILC2s. It is important to note that the proliferative capacity of ILC2s in the lungs upon a worm challenge remained unaffected in PD-L1-deficient mice (35). However, the release of ILC2 proliferative inhibition upon PD-L1 neutralization suggests the importance of PD-L1 signaling in ILC2 expansion *in vitro*, most likely *via* IFN-γ (**Figures 3E, G**). The mechanisms by which MAIT cells repress the functions of ILC2s and their proliferation are similar to those by NK cells. NK cells alleviate lung inflammation by suppressing the functions and proliferation of ILC2s through IFN-γ (26). Although NK cells and ILC2s belong to the family of innate lymphoid cells, the present results revealed that MAIT cells, innate-like T cells, inhibited cytokine production by ILC2s and their proliferation. These compromised features in ILC2s may ultimately alleviate airway inflammation in Vα19 and NOG mice.

An analysis of peripheral blood and bronchial biopsy samples from asthmatic patients showed a lower number of MAIT cells than in healthy subjects (29, 36). The number of IFN-γ^+^ MAIT cells was also lower in bronchial biopsy samples from asthmatic patients (29). Moreover, children with more MAIT cells at 1 year of age were less likely to develop asthma accompanying by IFN-γ-producing CD4^+^T cells by 7 years of age (37). These findings corroborate our result showing the MAIT cell-mediated repression of type 2 inflammation. While IFN-γ was detected in BALF upon the adoptive transfer of ILC2s and MAIT cells in NOG mice, MAIT cells had not been pretreated with IL-12, IL-15, or IL-18, similar to the *in vitro* coculture (**Figures 3B**, **4B**). Importantly, previous studies demonstrated that type 1 lymphocytes and/or NK cells are the source of IFN-γ, both of which are absent in NOG mice (26, 38). Therefore, it is plausible that IFN-γ is directly induced by IL-33 in MAIT cells (39). Alternatively, it is also possible that IL-33 activates cells other than MAIT cells in NOG mice, which, in turn, induced the production of IFN-γ by MAIT cells. Therefore, further studies are needed to identify the cells or mediator(s) linking IL-33 and MAIT cell activation. This will provide insights into the mechanisms by which MAIT cells repress the functions of ILC2s *in vivo* and will contribute to the development of therapeutic interventions for diseases in which ILC2s play a crucial role, such as atopic dermatitis, asthma, colitis, fibrosis, Helminth infection, and obesity (40, 41).

## Data availability statement

RNA-sequencing data deposited within the DNA Data Bank of Japan (Accession: DRR397569-DRR397576).

## Ethics statement

The animal study was reviewed and approved by Internal Animal Ethics Committee at Dokkyo Medical University (permission number 1243).

## Author contributions

Conception and design: YS, CS, and HW. Experimental work: YH-K, YS, HW, CS, and YI. Analysis and interpretation: YS, HW, CS. Drafting the manuscript and managing study: YS, HW, and CS. All authors contributed to the article and approved the submitted version.

## Funding

The Science Research Promotion Fund 2018-2019 (The Promotion and Mutual Aid Corporation for Private Schools of Japan) and 21K19732 (JSPS KAKENHI) to HW and 17H03565 (JSPS KAKENHI) and 26430084 (JSPS KAKENHI) to CS. The funders have no role in interpretation of the data and in designing the experiments.

## Acknowledgments

We thank H. Kaneko, Y. Machida, and T. Tsukahara (Animal Facility, Dokkyo Medical University) for animal maintenance, M. Ohyama and Y. Murakami (Host Defense Division, Dokkyo Medical University) for help in animal experiments, Y. Nonaka for cell sorting, N. Oshima for help in histology (Center for Research Collaboration and Support, Dokkyo Medical University), and the NIH Tetramer Core Facility (Emory University, GA, USA) for unlabeled and APC- and BV421-labeled mMR1 tetramers.

## Conflict of interest

The authors declare that the research was conducted in the absence of any commercial or financial relationships that could be construed as a potential conflict of interest.

## Publisher’s note

All claims expressed in this article are solely those of the authors and do not necessarily represent those of their affiliated organizations, or those of the publisher, the editors and the reviewers. Any product that may be evaluated in this article, or claim that may be made by its manufacturer, is not guaranteed or endorsed by the publisher.

## Supplementary material

Supplementary Figure 1 Gating strategy for identifying cell subsets in BAL. **(A)** Gating strategy for macrophages, lymphocytes, eosinophils, and neutrophils. Macrophages, lymphocytes, eosinophils, and neutrophils are defined as SSC^high^CD11b^int^ cells, CD19^+^ and/or CD3^+^ cells, CD11b^high^Siglec F^+^ cells, and CD11b^high^Ly6G^+^ cells, respectively, among CD45^+^ cells in wild-type mice (WT, upper panels) and Vα19 mice (Vα19, lower panels). **(B)** Gating strategy for MAIT cells and ILC2s. MAIT cells are defined as CD45^+^CD3^+^ 5-OP-RU-loaded mouse MR1-tetramer^+^ cells (MAIT cells), while ILC2s are defined as Lineage^-^CD45^+^ST2^+^CD90.2^+^ cells (ILC2) in WT and Vα19 mice.

Supplementary Figure 2 MAIT cells in the lung. The frequency of MAIT cells in Vα19 and WT (C57BL/6) mouse lung is shown. The number in the right panels shows the percentage of MAIT cells [TCRβ^+^MR1 tet (5-OP-RU-loaded mouse MR1-tetramer)^+^] cells among CD3^+^ cells. Data are representative of 3 mice.

Supplementary Figure 3 IL-12p70 and IL-23 in BALF IL-12p70 and IL-23 in BALF harvested at the indicated day after *A. alternata* challenge were quantified with LegendPlex as described in Materials and Methods. WT(AA); wild type mouse challenged with *A. alternata*, Vα19AA; Vα19 mouse challenged with *A. alternata*

Supplementary Figure 4 Purity of ILC2s and MAIT cells for the experiments (ILC2s): Sort-purified ILC2s (Lineage ^neg^CD45^+^CD90.2^+^ST2^+^ cells) were cultured for 14 days as described in Materials and Methods, and the purity was checked before the pertinent experiments by the flow cytometer (Attune Nxt). The number in the panel shows the purity of ILC2s (ST2^+^CD90.2^+^) among live CD45^+^ cells (right column). (MAIT cell): Similarly, sort-purified MAIT cells (B220^-^F4/80^-^TCRβ^+^mMR1-tetramer^+^ cells) were cultured for overnight as described in Materials and Methods, and the purity was checked. The number in the panel shows the purity of MAIT cells (TCRβ^+^mMR1-tetramer^+^ cells) among B220^-^F4/80^-^ cells (right column).

Supplementary Figure 5 cyt-MAIT cells inhibiting ILC2 division. Upon the challenge with IL-33, CFSE-labeled ILC2s (ILC2) were left untreated (upper panel) or cocultured with cyt-MAIT cells (cyt-MAIT) (lower panel). Cell division by ILC2s was visualized with flow cytometry on days 0 and 4. The number shown indicates the ILC2 count harboring the specific intensity of CFSE on day 0 (blue) and day 4 (red). Representative data from three independent experiments are shown.

Supplementary Figure 6 Expression of molecules relevant to ILC2s ILC2s cultured alone in the absence and presence of IL-33 or co-cultured with naïve MAIT cells (MAIT), IL12-, IL-15, and IL-18-stimulated MAIT cells (cyt-MAIT), and 5-OP-RU-stimulated MAIT cells (5OR-MAIT) for 5 days were analyzed for the expression of the indicated molecules in ILC2s. Data are shown as the geometric mean fluorescent intensity (gMFI) with mean ± SD (n=4). **P*< 0.05, ** *P <*0.01, *** *P <*0.001, **** *P*<0.0001

Supplementary Figure 7 Localization of ILC2s in the lung. ILC2s in the NOG mouse lungs are identified with the surrogate markers as described in . Mice were challenged with IL-33 after the adoptive transfer of ILC2s as described in Materials and Methods. ILC2s (red or magenta) together with background staining of CD3^+^ cells (green) are shown within tissue sections representing the perivascular, submucosal, and peribronchial regions. a: artery, b: bronchus. Nuclei are stained with DAPI. Bars indicate 50 μm. Representative data from 4 mice are shown.

Supplementary Table 1 List of antibodies used in the study.

Supplementary Table 2 Table summarizing DEG between MAIT cells from different sources. Most significant genes related to the suppressive activity of MAIT cells are shown with Log2 (FC) and adjusted *P* values. Other significant DEG between the groups are listed.

## References

[1] Collaborators, GBD 2019 Diseases and Injuries Ärnlöv J. Global burden of 369 diseases and injuries in 204 countries and territories, 1990–2019: a systematic analysis for the global burden of disease study 2019. Lancet (2020) 396:1204–22. doi: 10.1016/S0140-6736(20)30925-9 PMC7567026 33069326

[2] Wenzel SE. Severe adult asthmas: Integrating clinical features, biology, and therapeutics to improve outcomes. Am J Respir Crit Care Med (2021) 203:809–21. doi: 10.1164/rccm.202009-3631CI PMC8017568 33326352

[3] Moro K Yamada T Tanabe M Takeuchi T Ikawa T Kawamoto H. Innate production of T(H)2 cytokines by adipose tissue-associated c-Kit(+)Sca-1(+) lymphoid cells. Nat (London) (2010) 463:540–4. doi: 10.1038/nature08636 20023630

[4] Licona-Limon P Kim LK Palm NW Flavell RA. TH2, allergy and group 2 innate lymphoid cells. Nat Immunol (2013) 14:536–42. doi: 10.1038/ni.2617 23685824

[5] Starkey MR McKenzie AN Belz GT Hansbro PM. Pulmonary group 2 innate lymphoid cells: surprises and challenges. Mucosal Immunol (2019) 12:299–311. doi: 10.1038/s41385-018-0130-4 30664706 PMC6436699

[6] Akdis CA Arkwright PD Brüggen M Busse W Gadina M Guttman-Yassky E. Type 2 immunity in the skin and lungs. Allergy (Copenhagen) (2020) 75:1582–605. doi: 10.1111/all.14318 32319104

[7] Nussbaum JC Van Dyken SJ von Moltke J Cheng LE Mohapatra A Molofsky AB. Type 2 innate lymphoid cells control eosinophil homeostasis. Nat (London) (2013) 502:245–8. doi: 10.1038/nature12526 PMC3795960 24037376

[8] Turner J Morrison PJ Wilhelm C Wilson M Ahlfors H Renauld J. IL-9–mediated survival of type 2 innate lymphoid cells promotes damage control in helminth-induced lung inflammation. J Exp Med (2013) 210:2951–65. doi: 10.1084/jem.20130071 PMC3865473 24249111

[9] Wilhelm C Hirota K Stieglitz B Van Snick J Tolaini M Lahl K. An IL-9 fate reporter demonstrates the induction of an innate IL-9 response in lung inflammation. Nat Immunol (2011) 12:1071–7. doi: 10.1038/ni.2133 PMC3198843 21983833

[10] Godfrey DI Uldrich AP McCluskey J Rossjohn J Moody DB. The burgeoning family of unconventional T cells. Nat Immunol (2015) 16:1114–23. doi: 10.1038/ni.3298 26482978

[11] Hinks TSC. Mucosal-associated invariant T cells in autoimmunity, immune-mediated diseases and airways disease. Immunology (2016) 148:1–12. doi: 10.1111/imm.12582 26778581 PMC4819138

[12] Lezmi G Leite-de-Moraes M. Invariant natural killer T and mucosal-associated invariant T cells in asthmatic patients. Front Immunol (2018) 9:1766. doi: 10.3389/fimmu.2018.01766 30105031 PMC6077286

[13] Sugimoto C Fujita H Wakao H. Mice generated with induced pluripotent stem cells derived from mucosal-associated invariant T cells. bioRxiv (2022) 2022.07.27.501791. doi: 10.1101/2022.07.27.501791 PMC10813358 38255242

[14] Sugimoto C Murakami Y Ishii E Fujita H Wakao H. Reprogramming and redifferentiation of mucosal-associated invariant T cells reveal tumor inhibitory activity. eLife (2022) 11:e70848. doi: 10.7554/eLife.70848 35379387 PMC8983048

[15] Wakao H Sugimoto C. Mouse MAIT-like cells and mouse rich in MAIT cells. (Japan: (WO2021085450) (Japan: Dokkyo Medical University (2022). Available at: https://worldwide.espacenet.com/patent/search/family/075714600/publication/WO2021085450A1?q=WO2021085450A1.

[16] Moro K Ealey KN Kabata H Koyasu S. Isolation and analysis of group 2 innate lymphoid cells in mice. Nat Protoc (2015) 10:792–806. doi: 10.1038/nprot.2015.047 25927389

[17] McMillan SJ Kearley J Campbell JD Zhu X Larbi KY Shipley JM. Matrix metalloproteinase-9 deficiency results in enhanced allergen-induced airway inflammation. J Immunol (2004) 172:2586–94. doi: 10.4049/jimmunol.172.4.2586 14764732

[18] Xie S Issa R Sukkar MB Oltmanns U Bhavsar PK Papi A. Induction and regulation of matrix metalloproteinase-12in human airway smooth muscle cells. Respir Res (2005) 6:148. doi: 10.1186/1465-9921-6-148 16359550 PMC1363355

[19] Gueders MM Hirst SJ Quesada-Calvo F Paulissen G Hacha J Gilles C. Matrix metalloproteinase-19 deficiency promotes tenascin-c accumulation and allergen-induced airway inflammation. Am J Respir Cell Mol Biol (2010) 43:286–95. doi: 10.1165/rcmb.2008-0426OC 19843707

[20] Stolarski B Kurowska-Stolarska M Kewin P Xu D Liew FY. IL-33 exacerbates eosinophil-mediated airway inflammation. J Immunol (2010) 185:3472–80. doi: 10.4049/jimmunol.1000730 20693421

[21] Louahed J Zhou Y Maloy WL Rani PU Weiss C Tomer Y. Interleukin 9 promotes influx and local maturation of eosinophils. Blood (2001) 97:1035–42. doi: 10.1182/blood.v97.4.1035 11159534

[22] Fu Y Wang J Zhou B Pajulas A Gao H Ramdas B. An IL-9-pulmonary macrophage axis defines the allergic lung inflammatory environment. Sci Immunol (2022) 7:eabi9768. doi: 10.1126/sciimmunol.abi9768 35179949 PMC8991419

[23] Nobs SP Pohlmeier L Li F Kayhan M Becher B Kopf M. GM-CSF instigates a dendritic cell–t-cell inflammatory circuit that drives chronic asthma development. J Allergy Clin Immunol (2021) 147:2118–2133.e3. doi: 10.1016/j.jaci.2020.12.638 33440200

[24] Salou M Legoux F Gilet J Darbois A du Halgouet A Alonso R. A common transcriptomic program acquired in the thymus defines tissue residency of MAIT and NKT subsets. J Exp Med (2019) 216:133–51. doi: 10.1084/jem.20181483 PMC6314520 30518599

[25] Wolterink R Kleinjan A van Nimwegen M Bergen I de Bruijn M Levani Y. Pulmonary innate lymphoid cells are major producers of IL-5 and IL-13 in murine models of allergic asthma. Eur J Immunol (2012) 42:1106–16. doi: 10.1002/eji.201142018 22539286

[26] Bi J Cui L Yu G Yang X Chen Y Wan X. NK cells alleviate lung inflammation by negatively regulating group 2 innate lymphoid cells. J Immunol (1950) 2017) 198:3336–44. doi: 10.4049/jimmunol.1601830 28275135

[27] Helou DG Shafiei-Jahani P Lo R Howard E Hurrell BP Galle-Treger L. PD-1 pathway regulates ILC2 metabolism and PD-1 agonist treatment ameliorates airway hyperreactivity. Nat Commun (2020) 11:3998. doi: 10.1038/s41467-020-17813-1 32778730 PMC7417739

[28] Taylor S Huang Y Mallett G Stathopoulou C Felizardo TC Sun M. PD-1 regulates KLRG1+ group 2 innate lymphoid cells. J Exp Med (2017) 214:1663–78. doi: 10.1084/jem.20161653 PMC5461001 28490441

[29] Hinks TSC Zhou X Staples KJ Dimitrov BD Manta A Petrossian T. Innate and adaptive T cells in asthmatic patients: Relationship to severity and disease mechanisms. J Allergy Clin Immunol (2015) 136:323–33. doi: 10.1016/j.jaci.2015.01.014 PMC4534770 25746968

[30] Lezmi G Abou-Taam R Garcelon N Dietrich C Machavoine F Delacourt C. Evidence for a MAIT-17–high phenotype in children with severe asthma. J Allergy Clin Immunol (2019) 144:1714–1716.e6. doi: 10.1016/j.jaci.2019.08.003 31425779

[31] Ye L Pan J Pasha MA Shen X D’Souza SS Fung ITH. Mucosal-associated invariant T cells restrict allergic airway inflammation. J Allergy Clin Immunol (2020) 145:1469–1473.e4. doi: 10.1016/j.jaci.2019.12.891 31874183 PMC7214121

[32] Duerr CU McCarthy CDA Mindt BC Rubio M Meli AP Pothlichet J. Type I interferon restricts type 2 immunopathology through the regulation of group 2 innate lymphoid cells. Nat Immunol (2016) 17:65–75. doi: 10.1038/ni.3308 26595887 PMC9135352

[33] Moro K Kabata H Tanabe M Koga S Takeno N Mochizuki M. Interferon and IL-27 antagonize the function of group 2 innate lymphoid cells and type 2 innate immune responses. Nat Immunol (2016) 17:76–86. doi: 10.1038/ni.3309 26595888

[34] Legoux F Salou M Lantz O. MAIT cell development and functions: the microbial connection. Immun (Cambridge Mass.) (2020) 53:710–23. doi: 10.1016/j.immuni.2020.09.009 33053329

[35] Schwartz C Khan AR Floudas A Saunders SP Hams E Rodewald H. ILC2s regulate adaptive Th2 cell functions *via* PD-L1 checkpoint control. J Exp Med (2017) 214:2507–21. doi: 10.1084/jem.20170051 PMC5584124 28747424

[36] Ishimori A Harada N Chiba A Harada S Matsuno K Makino F. Circulating activated innate lymphoid cells and mucosal-associated invariant T cells are associated with airflow limitation in patients with asthma. Allergol Int (2017) 66:302–9. doi: 10.1016/j.alit.2016.07.005 27575652

[37] Chandra S Wingender G Greenbaum JA Khurana A Gholami AM Ganesan A. Development of asthma in inner-city children: Possible roles of MAIT cells and variation in the home environment. J Immunol (1950) 2018) 200:1995–2003. doi: 10.4049/jimmunol.1701525 PMC5840005 29431692

[38] Cautivo KM Matatia PR Lizama CO Mroz NM Dahlgren MW Yu X. Interferon gamma constrains type 2 lymphocyte niche boundaries during mixed inflammation. Immun (Cambridge Mass.) (2022) 55:254–271.e7. doi: 10.1016/j.immuni.2021.12.014 PMC8852844 35139352

[39] Azzout M Dietrich C Machavoine F Gastineau P Bottier A Lezmi G. IL-33 enhances IFNγ and TNFα production by human MAIT cells: A new pro-Th1 effect of IL-33. Int J Mol Sci (2021) 22:10602. doi: 10.3390/ijms221910602 34638950 PMC8508606

[40] Kato A. Group 2 innate lymphoid cells in airway diseases. Chest (2019) 156:141–9. doi: 10.1016/j.chest.2019.04.101 PMC7118243 31082387

[41] Halim TYF. Group 2 innate lymphoid cells in disease. Int Immunol (2016) 28:13–22. doi: 10.1093/intimm/dxv050 26306498 PMC5891987

