# Supplementary figures and images for: Mucosal-associated invariant T cells repress group 2 innate lymphoid cells in Alternaria alternata-induced model of allergic airway inflammation
Source: Front Immunol. 2022 Nov 15;13:1005226. doi: 10.3389/fimmu.2022.1005226 (PMC9706205; doi:10.3389/fimmu.2022.1005226)

**A**

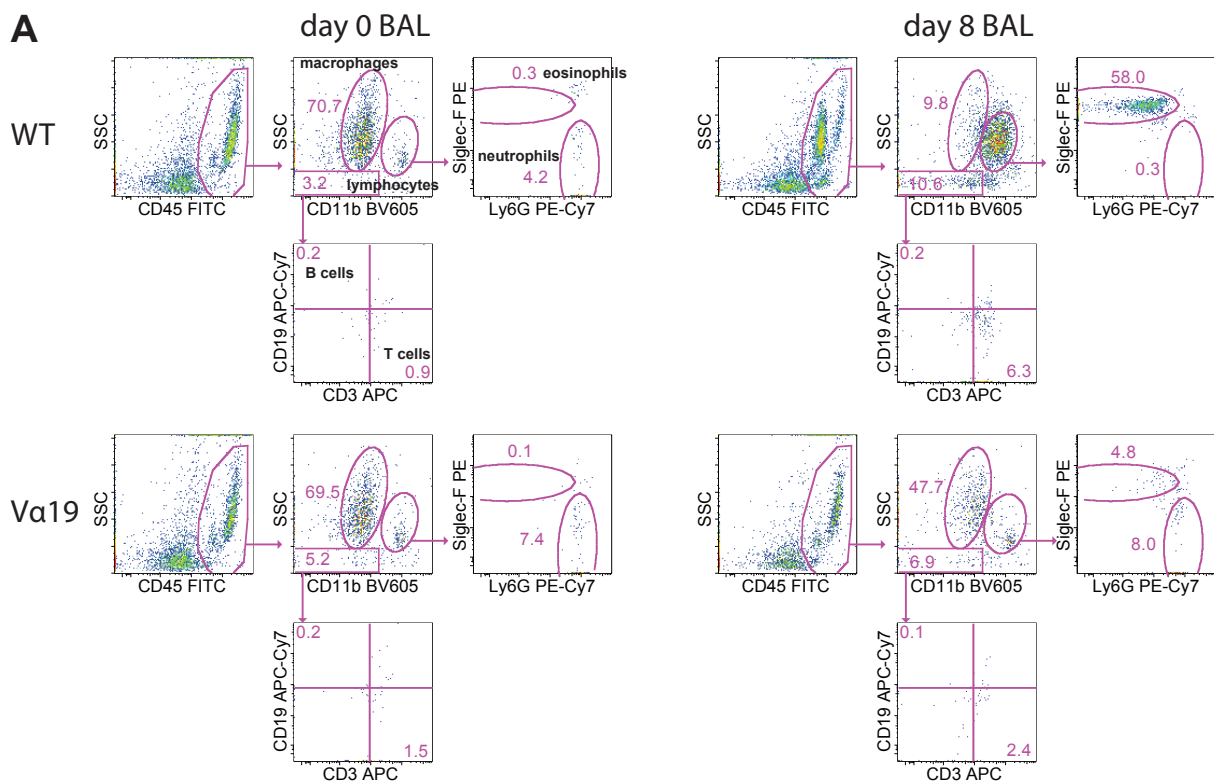

**B**

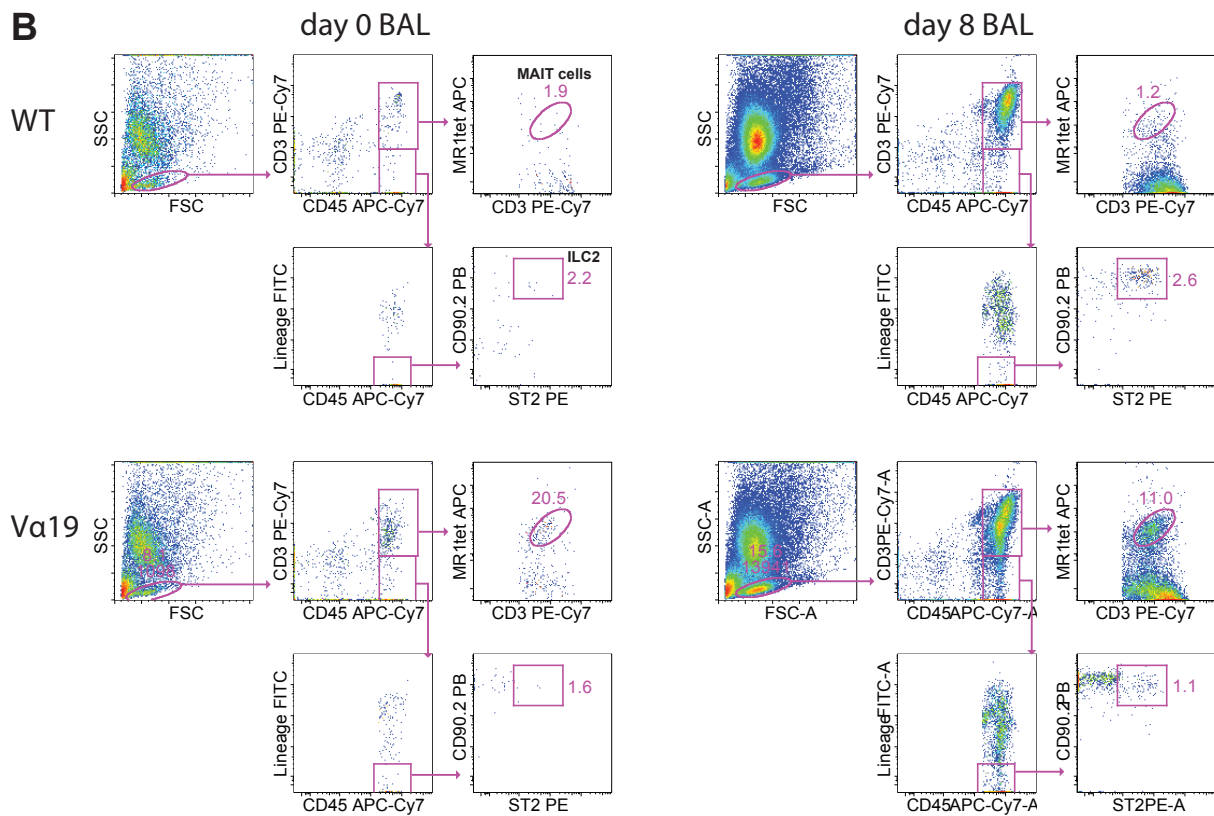

Supplement: Supplementary Figure 1 — Gating strategy for identifying cell subsets in BAL. (A) Gating strategy for macrophages, lymphocytes, eosinophils, and neutrophils. Macrophages, lymphocytes, eosinophils, and neutrophils are defined as SSChighCD11bint cells, CD19+ and/or CD3+ cells, CD11bhighSiglec F+ cells, and CD11bhighLy6G+ cells, respectively, among CD45+ cells in wild-type mice (WT, upper panels) and Vα19 mice (Vα19, lower panels). (B) Gating strategy for MAIT cells and ILC2s. MAIT cells are defined as CD45+CD3+ 5-OP-RU-loaded mouse MR1-tetramer+ cells (MAIT cells), while ILC2s are defined as Lineage-CD45+ST2+CD90.2+ cells (ILC2) in WT and Vα19 mice. [file DataSheet_1.pdf]

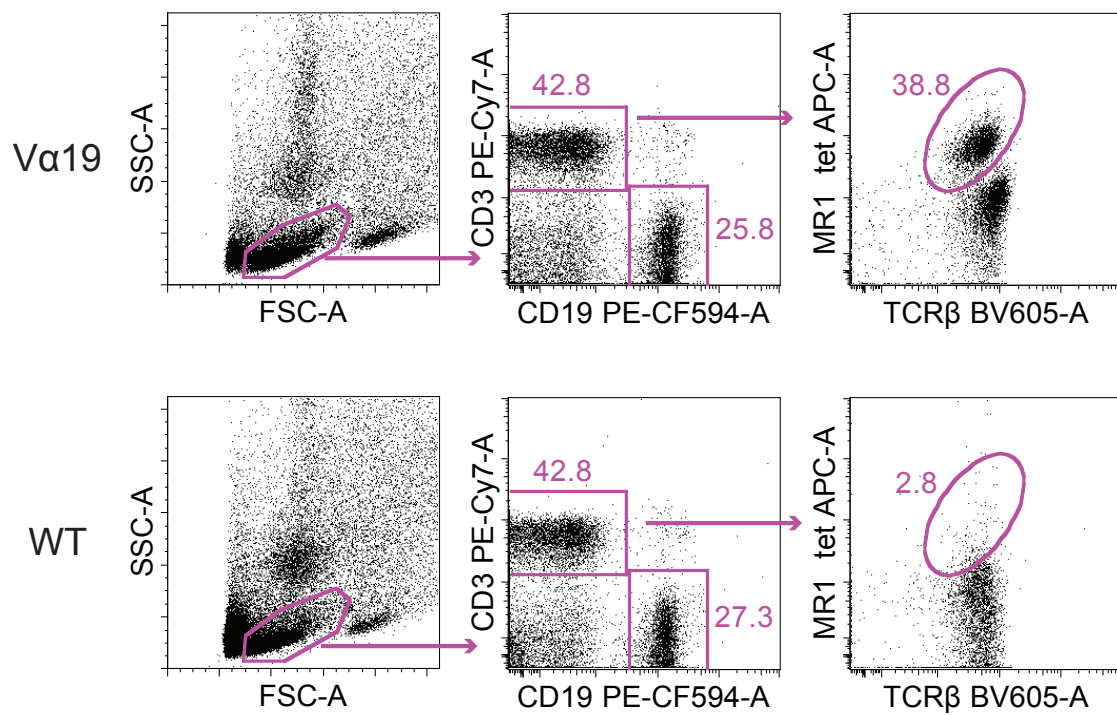

Supplement: Supplementary Figure 2 — MAIT cells in the lung. The frequency of MAIT cells in Vα19 and WT (C57BL/6) mouse lung is shown. The number in the right panels shows the percentage of MAIT cells [TCRβ+MR1 tet (5-OP-RU-loaded mouse MR1-tetramer)+] cells among CD3+ cells. Data are representative of 3 mice. [file DataSheet_2.pdf]

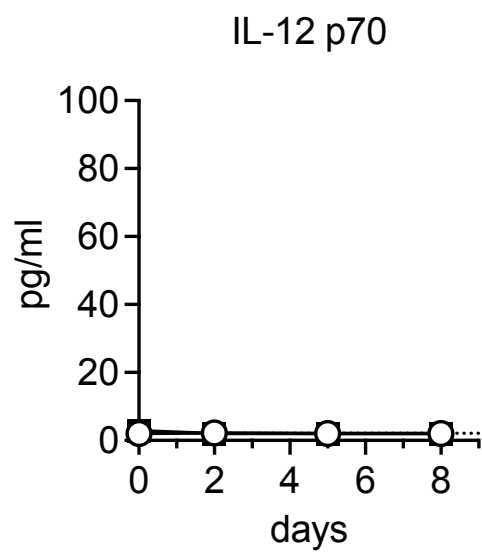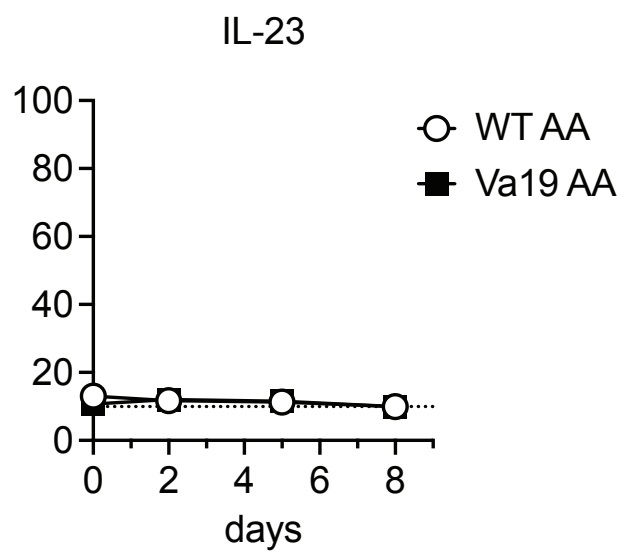

Supplement: Supplementary Figure 3 — IL-12p70 and IL-23 in BALF IL-12p70 and IL-23 in BALF harvested at the indicated day after A. alternata challenge were quantified with LegendPlex as described in Materials and Methods. WT(AA); wild type mouse challenged with A. alternata, Vα19AA; Vα19 mouse challenged with A. alternata [file DataSheet_3.pdf]

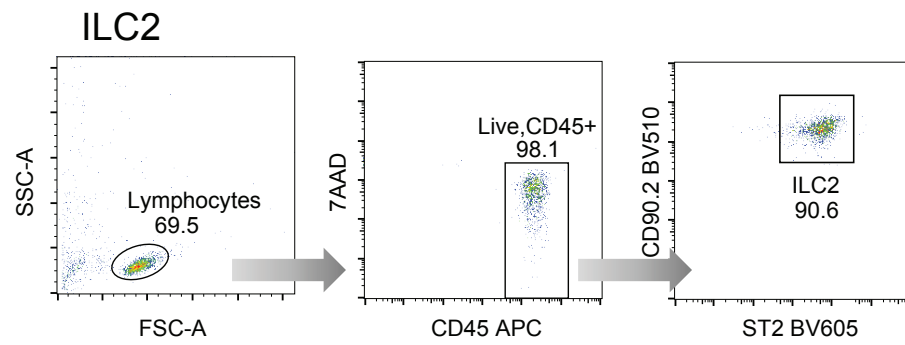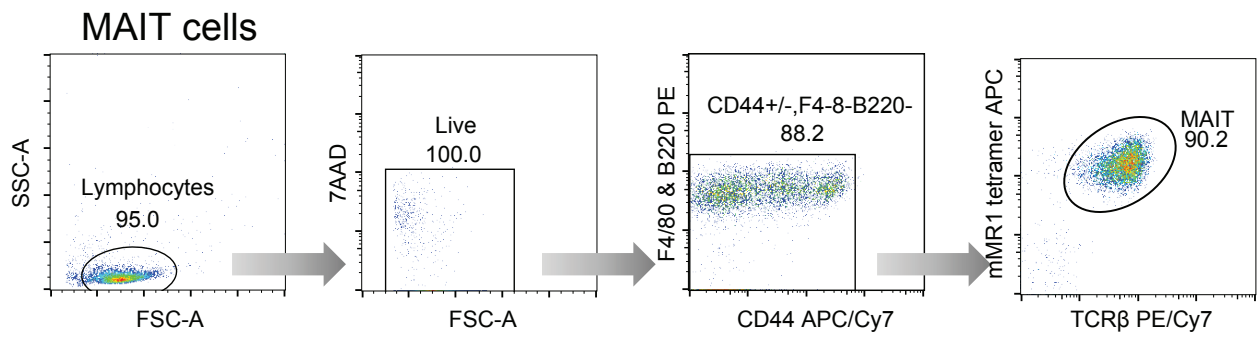

Supplement: Supplementary Figure 4 — Purity of ILC2s and MAIT cells for the experiments (ILC2s): Sort-purified ILC2s (Lineage negCD45+CD90.2+ST2+ cells) were cultured for 14 days as described in Materials and Methods, and the purity was checked before the pertinent experiments by the flow cytometer (Attune Nxt). The number in the panel shows the purity of ILC2s (ST2+CD90.2+) among live CD45+ cells (right column). (MAIT cell): Similarly, sort-purified MAIT cells (B220-F4/80-TCRβ+mMR1-tetramer+ cells) were cultured for overnight as described in Materials and Methods, and the purity was checked. The number in the panel shows the purity of MAIT cells (TCRβ+mMR1-tetramer+ cells) among B220-F4/80- cells (right column). [file DataSheet_4.pdf]

ILC2 only

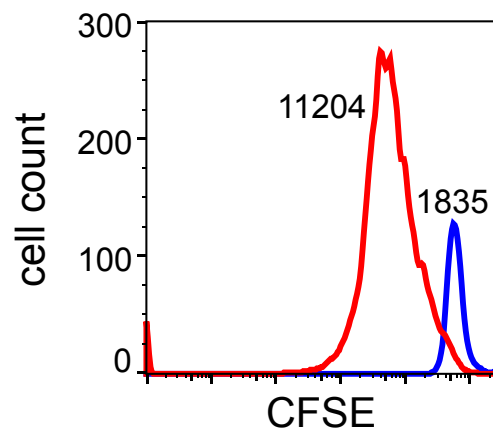

day 0  
day 4

ILC2  
+cyt-MAIT

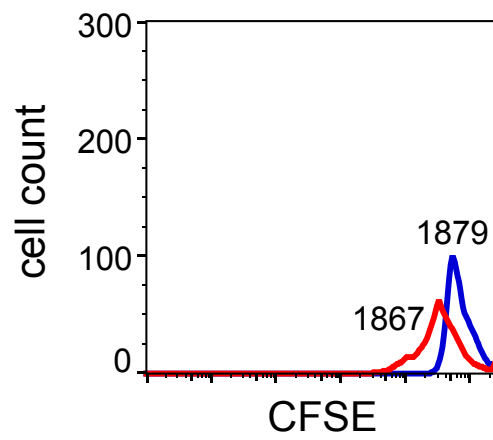

Supplement: Supplementary Figure 5 — cyt-MAIT cells inhibiting ILC2 division. Upon the challenge with IL-33, CFSE-labeled ILC2s (ILC2) were left untreated (upper panel) or cocultured with cyt-MAIT cells (cyt-MAIT) (lower panel). Cell division by ILC2s was visualized with flow cytometry on days 0 and 4. The number shown indicates the ILC2 count harboring the specific intensity of CFSE on day 0 (blue) and day 4 (red). Representative data from three independent experiments are shown. [file DataSheet_5.pdf]

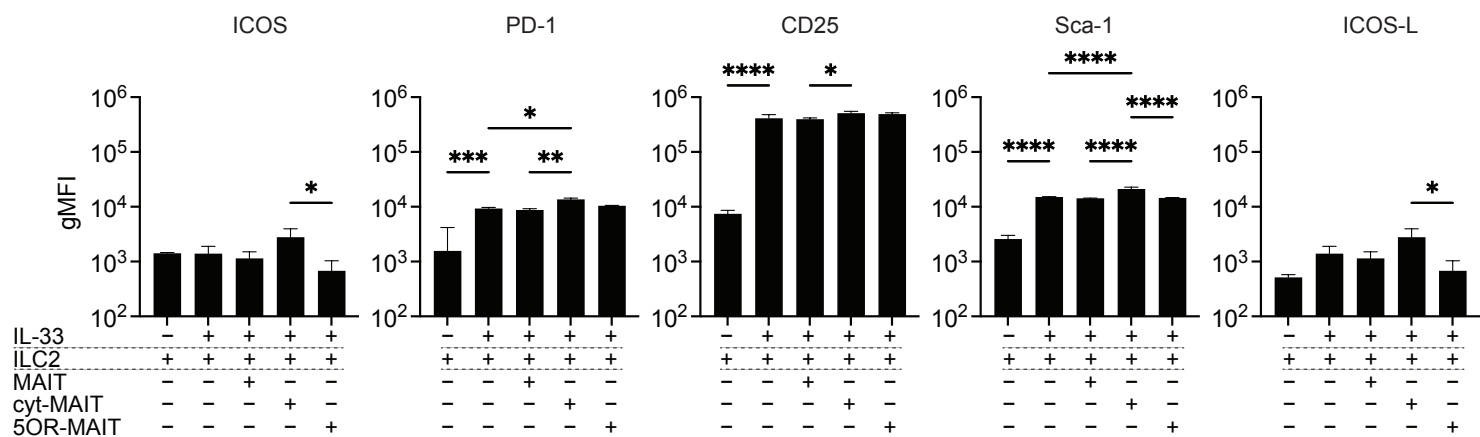

Supplement: Supplementary Figure 6 — Expression of molecules relevant to ILC2s ILC2s cultured alone in the absence and presence of IL-33 or co-cultured with naïve MAIT cells (MAIT), IL12-, IL-15, and IL-18-stimulated MAIT cells (cyt-MAIT), and 5-OP-RU-stimulated MAIT cells (5OR-MAIT) for 5 days were analyzed for the expression of the indicated molecules in ILC2s. Data are shown as the geometric mean fluorescent intensity (gMFI) with mean ± SD (n=4). *P< 0.05, ** P <0.01, *** P <0.001, **** P<0.0001 [file DataSheet_6.pdf]

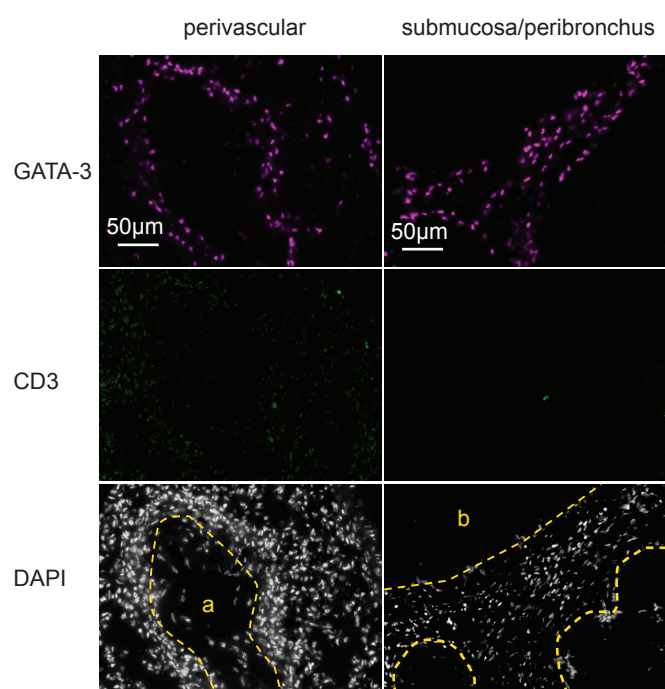

Supplement: Supplementary Figure 7 — Localization of ILC2s in the lung. ILC2s in the NOG mouse lungs are identified with the surrogate markers as described in . Mice were challenged with IL-33 after the adoptive transfer of ILC2s as described in Materials and Methods. ILC2s (red or magenta) together with background staining of CD3+ cells (green) are shown within tissue sections representing the perivascular, submucosal, and peribronchial regions. a: artery, b: bronchus. Nuclei are stained with DAPI. Bars indicate 50 μm. Representative data from 4 mice are shown. [file DataSheet_7.pdf]
